# Potential Binding Sites of Pharmacological Chaperone NCGC00241607 on Mutant β-Glucocerebrosidase and Its Efficacy on Patient-Derived Cell Cultures in Gaucher and Parkinson’s Disease

**DOI:** 10.3390/ijms24109105

**Published:** 2023-05-22

**Authors:** Alena E. Kopytova, George N. Rychkov, Alexander A. Cheblokov, Elena V. Grigor’eva, Mikhail A. Nikolaev, Elena S. Yarkova, Diana A. Sorogina, Farid M. Ibatullin, Galina V. Baydakova, Artem D. Izyumchenko, Daria A. Bogdanova, Vitali M. Boitsov, Akim V. Rybakov, Irina V. Miliukhina, Vadim A. Bezrukikh, Galina N. Salogub, Ekaterina Y. Zakharova, Sofya N. Pchelina, Anton K. Emelyanov

**Affiliations:** 1 Petersburg Nuclear Physics Institute Named by B.P. Konstantinov of National Research Center «Kurchatov Institute», Gatchina 188300, Russia; kopytovaalena@mail.ru (A.E.K.);; 2 Department of Molecular Genetic and Nanobiological Technologies, Pavlov First Saint-Petersburg State Medical University, Saint-Petersburg 197022, Russia; 3 Institute of Biomedical Systems and Biotechnology, Peter the Great St.Petersburg Polytechnic University, Saint-Petersburg 195251, Russia; 4 Institute of Cytology and Genetics Siberian Branch of Russian Academy of Sciences, Novosibirsk 630090, Russia; 5 Meshalkin National Medical Research Center, Ministry of Health of the Russian Federation, Novosibirsk 630055, Russia; 6 Research Centre for Medical Genetics, Moscow 115522, Russia; 7 Laboratory of Nanobiotechnology, Saint-Petersburg National Research Academic University of the Russian Academy of Sciences, Saint-Petersburg 194021, Russia; 8 N.P. Bechtereva Institute of the Human Brain RAS, Saint-Petersburg 197376, Russia; 9 Almazov National Medical Research Centre, Saint-Petersburg 197341, Russia

**Keywords:** pharmacological chaperones, Parkinson’s disease, Gaucher disease, glucocerebrosidase, non-inhibitory chaperones, allosteric binding site, binding free energy, iPSC-derived dopaminergic neurons

## Abstract

Mutations in the *GBA1* gene, encoding the lysosomal enzyme glucocerebrosidase (GCase), cause Gaucher disease (GD) and are the most common genetic risk factor for Parkinson’s disease (PD). Pharmacological chaperones (PCs) are being developed as an alternative treatment approach for GD and PD. To date, NCGC00241607 (NCGC607) is one of the most promising PCs. Using molecular docking and molecular dynamics simulation we identified and characterized six allosteric binding sites on the GCase surface suitable for PCs. Two sites were energetically more preferable for NCGC607 and located nearby to the active site of the enzyme. We evaluated the effects of NCGC607 treatment on GCase activity and protein levels, glycolipids concentration in cultured macrophages from GD (*n* = 9) and GBA-PD (*n* = 5) patients as well as in induced human pluripotent stem cells (iPSC)—derived dopaminergic (DA) neurons from GBA-PD patient. The results showed that NCGC607 treatment increased GCase activity (by 1.3-fold) and protein levels (by 1.5-fold), decreased glycolipids concentration (by 4.0-fold) in cultured macrophages derived from GD patients and also enhanced GCase activity (by 1.5-fold) in cultured macrophages derived from GBA-PD patients with N370S mutation (*p* < 0.05). In iPSC-derived DA neurons from GBA-PD patients with N370S mutation NCGC607 treatment increased GCase activity and protein levels by 1.1-fold and 1.7-fold (*p* < 0.05). Thus, our results showed that NCGC607 could bind to allosteric sites on the GCase surface and confirmed its efficacy on cultured macrophages from GD and GBA-PD patients as well as on iPSC-derived DA neurons from GBA-PD patients.

## 1. Introduction

Gaucher disease (GD) is the most common of the lipid storage disorder, caused by *GBA1* mutations, resulting in the deficiency of the lysosomal enzyme glucocerebrosidase (GCase) activity and the accumulation of the lipid substrates glucosylceramide (GlcCer) and glucosylsphingosine (GlcSph) within the lysosomes of macrophages [1,2]. Clinical manifestations of the most common form of GD (nonneuropathic, type 1) include enlarged spleen and liver, platelet deficiency, anemia and bone disease. Types 2 and 3 GD are neuronopathic forms [2]. More than 480 mutations in *GBA1* gene are reported for GD [3]. The frequency of *GBA1* mutations varies among different ethnic groups. The most frequent *GBA1* variant in Ashkenazi Jews and European populations is N370S while in some East and South Asian populations it is L444P [4,5,6]. Homozygosity for N370S mutation causes nonneuropathic GD, whereas homozygosity for L444P mutation is associated with a severe neuronopathic disease manifestation [4,7,8,9].

*GBA1* mutations are the major genetic risk factor for Parkinson’s Disease (PD) (5–20% of PD cases) and may increase PD risk by up to 10 times. PD is a common neurodegenerative disorder characterized by a progressive loss of dopaminergic neurons in the substantia nigra pars compacta and intracytoplasmic inclusions predominantly composed of aggregated alpha-synuclein [1,10,11,12,13,14,15,16]. At present, PD therapy is symptomatic without affecting the disease progression [10]. Understanding the relationship between GCase and alpha-synuclein could lead to the development of novel therapeutic approaches for patients with PD associated with mutations in the *GBA1* gene (GBA-PD).

Current treatments for GD are enzyme replacement therapy (ERT) and substrate reduction therapy (SRT). Nevertheless, both strategies are not effective in neuronopathic forms of GD [17,18]. Pharmacological chaperones (PCs) of GCase could successfully cross the blood-brain barrier and might modulate GCase activity and protein levels as well as GCase translocation to the lysosome and could be an alternative therapy not only for GD (including neuronopathic forms), but also for GBA-PD [13,19].

One of the most promising GCase PC with unique chaperone characteristics is a well-known mucolytic agent ambroxol [18,19,20,21,22,23]. Previously we constructed an atomistic model of mutant form of GCase and confirmed that ambroxol is a mixed-type enzyme inhibitor [24].

As known, non-inhibitory chaperones can facilitate GCase folding and assist it to translocate into lysosomes without interfering with the active center of the protein and thus can restore the enzyme activity. Promising GCase non-inhibitory PCs NCGC00241607 (NCGC607) and NCGC00188758 have been uncovered and investigated in vitro for GD1, GD2 and also for PD [18,25,26,27,28,29,30,31]. However, binding sites on the enzyme surface for these PCs have not yet been identified. Nowadays studies aimed at finding new non-inhibiting PCs, as well as descriptions of the mechanism of action of the already described non-inhibiting PCs are extremely relevant.

Here, for the first time, using molecular docking and molecular dynamics simulations we investigated the allosteric sites of GCase that could incorporate NCGC607 compound described previously by the group of Ellen Sidransky [25,27]. Also for the first time we assessed efficiency of NCGC607 treatment on GCase activity and protein levels, as well as glycolipids concentration in peripheral blood mononuclear cells (PBMC)—derived macrophages (cultured macrophages) from patients with GD and GBA-PD with different *GBA1* mutations and in iPSCs-derived dopaminergic (DA) neurons from GBA-PD patient.

## 2. Results

### 2.1. Binding Sites Description

**Figure 1:**
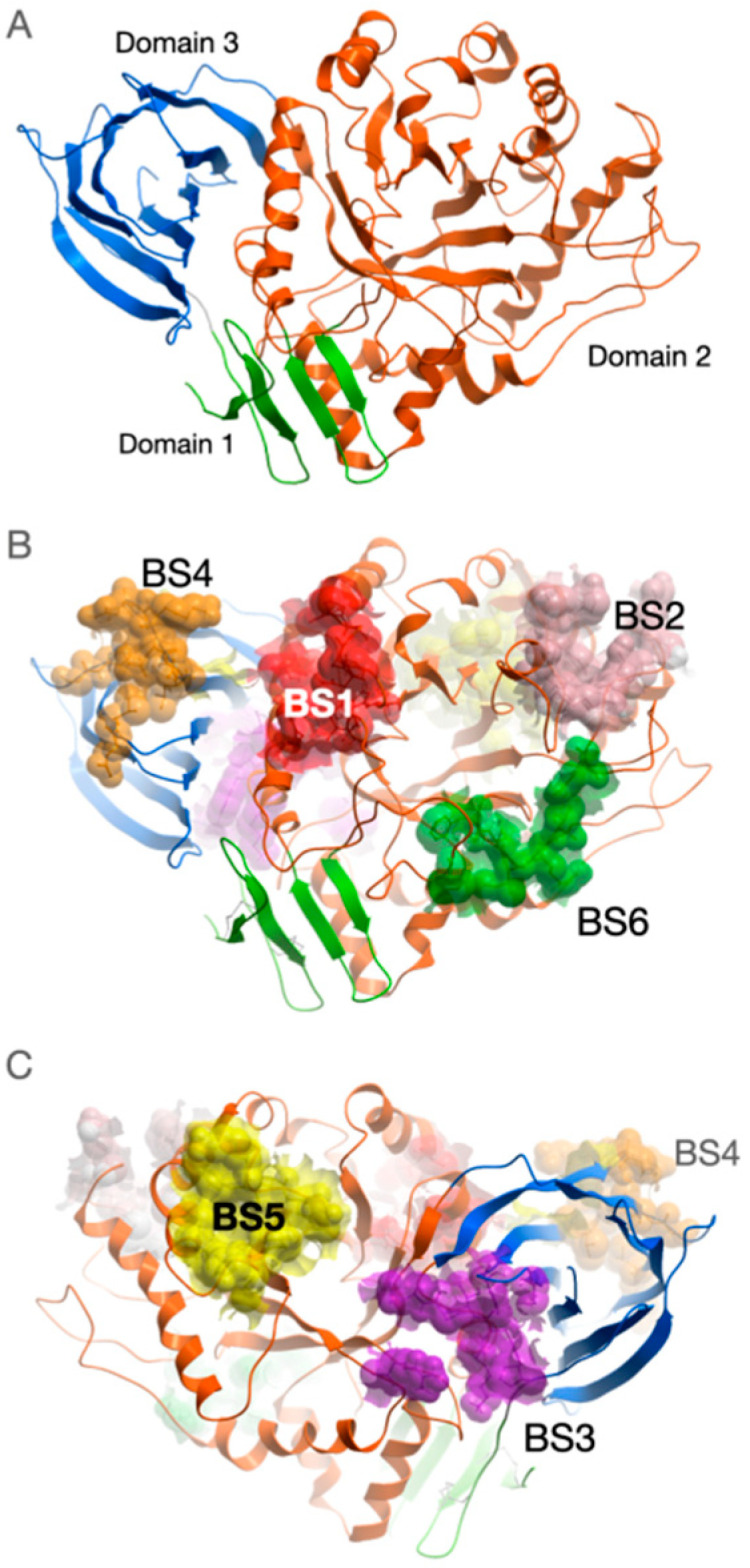
Spatial structure of GCase with potential binding sites mapped on its surface. (**A**) GCase comprises three domains marked by different colors: green—Domain 1, orange—Domain 2, blue—Domain 3. Amino acid residues combined into potential binding sites (BS) are displayed by van der Waals spheres: (**B**) view from the side of GCase where the active site is located (red—BS1, pink—BS2, green—BS6, orange—BS4), (**C**) view from the opposite side of protein (violet—BS3, orange—BS4, yellow—BS5). The model of the mutant form of the enzyme was constructed by introducing the amino acid substitution N370S into the structure deposited in 2NT1 PDB entry (subunit A) followed by relaxation using molecular dynamics simulations in explicit water.

GCase consists of three discontinuous domains (Figure 1A) and its structural aspects are discussed in details elsewhere [32].

Along with the active center, we identified six potential allosteric binding sites on the surface of GCase (Figure 1B,C). Binding sites were enumerated according to their enclosed volume. Amino acid residues that form these binding sites are listed in Table 1. For each site, the lowest binding free energy of NCGC607 found among the analyzed fragments of the trajectories in which the complex with GCase remained stable is given.

Four of six predicted binding sites are located within Domain 2 close to active center (BS1, BS2, BS5 and BS6). BS1 is the largest one and it is enclosed by Loops 6 (residues 311–319) and 7 (residues 345–349) as well as by alpha-helix 7 (residues 357–371). Site BS2 is enclosed by alpha-helices 3 and 4 and their N-flanking loops (residues 240–259 and 193–209). Site BS5 is located in the cleft formed by alpha-helices 4 and 5 and their C-flanking regions and N-terminal residues of beta-strand 5 (residues 265–277 and 303–306). Site BS6 is formed by Loops 2 (residues 127–132) and 8 (residues 390–393) as well as by alpha-helix 1a (residues 88–92).

Sites BS3 and BS4 are located in Domain 3. The majority of residues forming the BS3 site belong to loop (425–431) connecting Domain 2 and beta-strand 4 of the Domain 3 and loop (451–453) connecting beta-strands 5 and 6 of Domain 3. BS4 site is predominantly formed by residues from another loop (439–444) connecting beta-strands 3 and 4 as well as loop connecting beta-strands 5 and 6 of Domain 3.

Inversely to sites BS1, BS2, BS4 and BS6, binding sites BS3 and BS5 are located on the protein surface on the opposite side of the active center.

In the identified sites we analyzed binding modes of NCGC607 compound in terms of binding free energy and mobility of the compound during 50 ns molecular dynamics (MD) simulations. In the case then different poses of compound are characterized by similar binding free energy values, we considered the less mobile pose as preferential.

### 2.2. NCGC607

**Figure 2:**
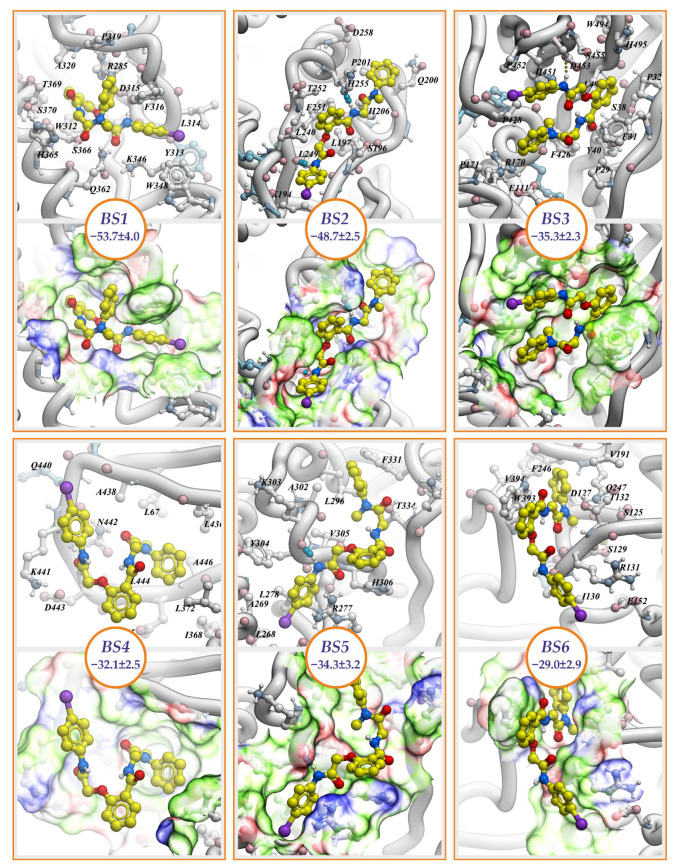
Binding modes of NCGC607 compound. Poses having the lowest values of binding free energies are shown. Compound and interacting with it amino acid residues are in a balls-and-sticks representation. Hydrogen bonds are depicted by dotted lines. The molecular surface is colored according to binding properties of specific chemical groups within amino acid residues forming the pockets: hydrogen bond donors (blue), hydrogen bond acceptors (red), and hydrophobic groups (green). Circles hold values of NCGC607 binding free energy measured in kcal/mol.

Compound NCGC607 forms the most stable complex with GCase when it binds at the BS1 site. Here, two poses of the compound have the minimal values of binding free energies: BS1.1 (−53.7 ± 4.0 kcal/mol) (Figure 2, BS1) and BS1.2 (−50.2 ± 3.1 kcal/mol) (Supplementary Figure S1). In BS1.1 pose, NCGC607 has U-shape conformation where N-methylaniline group takes up position perpendicular to the plane in which the rest of the molecule lies. Its benzamide group penetrates to the cavity appeared on the GCase surface in a result of N370S amino acid substitution. N-methylaniline group interacts with Loop 6. This pose can be stabilized by 5 hydrogen bonds between: N atom of 4-iodoaniline group and Oδ Asp315, N atom of benzamide group and Oγ Ser366, O atom of oxoethyl group and Nε Gln362, O atom of oxoethoxy group and Nε Gln362, O atom of oxoethoxy group and Nζ Lys346. Nevertheless, exposed to the solvent N-methylaniline group displayed high mobility during MD simulations, but its position can be stabilized by flexible hydrophobic residue Phe316, the interaction with which significantly (about 8 kcal/mol) decreases the binding free energy of the compound. On the other hand, along MD trajectory NCGC607 stably keeps BS1.2 pose in binding site. In this pose N-methylaniline group is buried into the cavity close to S370 residue and 4-iodoaniline group is buried into a cavity enclosed by Loops 6 and 7 and alpha-helix 7, benzamide group interacts with Loop 6. The compound can form hydrogen bonds by N atoms of benzamide and 4-iodoaniline groups with O atom of F316.

NCGC607 effectively hides its hydrophobic surface by expanding into a linear conformation in the long cleft of the BS2 site (Figure 2, BS2). The compound is oriented in the site in such a way that the N-methylaniline group is near the N-terminus of alpha-helices 3 and 4. This pose is characterized by binding free energy −48.7 ± 2.5 kcal/mol and NCGC607 compound remained practically inflexible throughout the entire MD trajectory. Compound can be stabilized by five possible hydrogen bonds between: O atom of oxoethyl group and Nε atom of His206, O atom of benzamide group and N Leu197 or Oγ Ser196, O atom of oxoethoxy group and Oγ Ser196, N atom of 4-iodoaniline group and O Leu249, N atom of benzamide group and Nδ atom of His255.

In BS3 site, BS3.1 pose had the lowest binding free energy (−35.3 ± 2.3 kcal/mol) and stably kept its position. Here, U-shaped conformation of NCGC607 resembles that in BS1.2 pose. Benzamide group is buried into the cavity, while 4-iodoaniline and N-methylaniline groups are sticked out to solvent. The compound forms only one hydrogen bond by N atom of 4-iodoaniline group with Oδ1 or Oδ2 atom of Asp453.

In BS4, BS5 and BS6 sites, preferential poses of NCGC607 compound in terms of binding free energies are characterized by its higher values: −32.1 ± 2.5 kcal/mol, −34.3 ± 3.2 kcal/mol and −29.0 ± 2.9 kcal/mol correspondingly. Only in one of the three independent runs of MD simulations, NCGC607 held a stable position in the BS4 site, taken a shape of letter S. The topology of the site does not allow the compound to immerse into a surface of the protein. NCGC607 can form three possible hydrogen bonds with the protein between: O atom of oxoethoxy group and N atom of Asn442 or N atom of Asp443, O atom of oxoethyl group and Nδ Asn442. In BS5 site in preferential conformation NCGC607 spans the space between C-terminal regions of alpha-helices 4, 5 and 6, so that 4-iodoaniline group is covered by the loop flanking C-termini of alpha-helix 4. Single hydrogen bond is formed in this complex between N atom of 4-iodoaniline group and O atom of Lys303. Biding properties of BS6 site towards NCGC607 are the weakest. In each of the three MD runs, the chemical compound significantly changed its conformation relative to the initial one. In the most energetically favorable pose of NCGC607, N-methylanilin group is immersed in the cleft between loops 2, 3, 4 and 8, while the position of the benzamide group is stabilized by the W393 amino acid residue. There are no hydrogen bonds between the compound and the protein.

### 2.3. The Effects of NCGC607 Treatment in Cultured Macrophages and iPSC-Derived DA Neurons from GD and GBA-PD Patients

In our study cultured macrophages and iPSC-derived DA neurons were used to evaluate the effects of NCGC607 on the restoration of GCase activity, GCase levels and hexosylsphingosine (HexSph) concentration in GD and GBA-PD patients with various severity of *GBA1* mutations and controls. Mutation N370S was regarded as “mild” and L444P, c84dupG, R120W and W184R as “severe” [4,33].

As we previously shown cultured macrophages reflected alterations in GCase activity level and HexSph concentration in peripheral blood [24].

### 2.4. The Effects of NCGC607 Treatment in Cultured Macrophages from GD and GBA-PD Patients

#### 2.4.1. GCase Activity and Protein Levels

**Figure 3:**
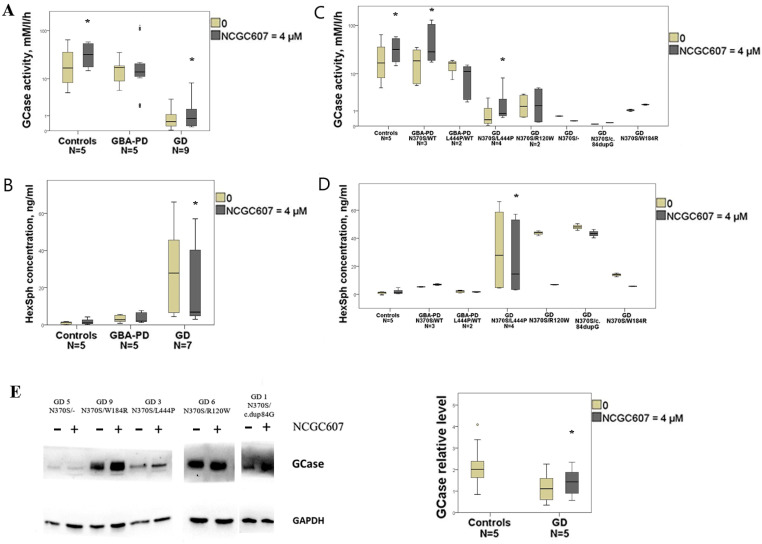
Evaluation of the NCGC607 effect (4 µM, 4 days) on GCase activity and protein levels, HexSph concentration in cultured macrophages from GD, GBA-PD patients and controls. (**A**) GCase activity (log scale) and (**B**) HexSph concentration in cultured macrophages from GD, GBA-PD patients and controls. (**C**) GCase activity (log scale) and (**D**) HexSph concentration in GD and GBA-PD macrophages depending on the type of *GBA1* mutations. (**E**) Western blot analysis of GCase levels in GD macrophages. * *p*-value < 0.05 compared to untreated cells.

In GD cultured macrophages treated with NCGC607 GCase activity was increased by 1.3-fold (*p* = 0.003) (Figure 3A). Particularly, in cultured macrophages from GD patients with genotype N370S/L444P (GD2, GD3, GD4 and GD7) GCase activity was enhanced by 1.4-fold (*p* = 0.017) (Figure 3C). We observed an individual effect of NCGC607 treatment on GCase activity restoration for each patient, (Supplementary Figure S2a), but the differences did not reach statistical significance (*p* > 0.05).

In GBA-PD (3 patients with N370S/WT mutation and 2 patients with L444P/WT) cultured macrophages NCGC607 treatment did not affect GCase activity (*p* > 0.05) (Figure 3A). At the same time, NCGC607 treatment of cultured macrophages derived from GBA-PD patients with genotype N370S/WT (GBA-PD3, GBA-PD4, GBA-PD5), but not L444P/WT (GBA-PD1, GBA-PD2) resulted in increased GCase activity by 1.5-fold (*p* = 0.008) (Figure 3C).

Notably, after NCGC607 treatment GCase activity was also increased in cultured macrophages derived from controls by 2.1-fold (*p* = 0.034) (Figure 3A).

In the presence of NCGC607 the ratio of GCase to GAPDH in GD cultured macrophages was increased in 1.5-fold (*p* = 0.003) (Figure 3E).

#### 2.4.2. HexSph Concentration

In GD cultured macrophages treated with NCGC607 the significant reduction of HexSph concentration by 4.0-fold was observed (*p* < 0.0001) (Figure 3B). Particularly in cultured macrophages derived from GD patients with genotype N370S/L444P (GD2, GD3, GD4 and GD7) NCGC607 treatment decreased HexSph concentration by 1.9-fold (*p* = 0.008) (Figure 3D). In addition, we observed an individual effect of NCGC607 treatment on reduction of HexSph concentration for each patient, but the differences did not reach statistical significance (*p* > 0.05) (Supplementary Figure S2b).

We observed no differences in HexSph concentration in GBA-PD cultured macrophages after NCGC607 treatment (*p* > 0.05) (Figure 3B,D). 

### 2.5. The Effects of NCGC607 Treatment in iPSC-Derived DA Neurons from GBA-PD Patient

Previously obtained, characterized and registered in the Human Pluripotent Stem Cell Registry hPSCreg (https://hpscreg.eu; accessed on 19 May 2023) iPSC lines were used for differentiation to DA neurons.

Three iPSC lines obtained from one PD patient with a mutation in the *GBA1* gene (https://hpscreg.eu/cell-line/ICGi034-A; https://hpscreg.eu/cell-line/ICGi034-B; https://hpscreg.eu/cell-line/ICGi034-C; all accessed on 19 May 2023) and two apparently healthy individuals (https://hpscreg.eu/cell-line/ICGi021-A; https://hpscreg.eu/cell-line/ICGi022-A; all accessed on 19 May 2023) were taken into the study.

**Figure 4:**
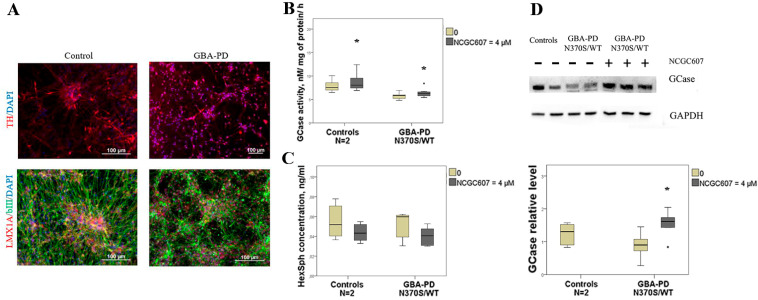
Evaluation of the NCGC607 effect (4 µM, 21 days) on GCase activity and protein levels, HexSph concentration in iPSC-derived DA neurons from GBA-PD patient (N370S/WT) and controls. (**A**) Immunofluorescent staining of DA neurons for neuronal markers. (**B**) GCase activity and (**C**) HexSph concentration in DA neurons from patient with GBA-PD (N370S/WT) and controls. (**D**) Western blot analysis of GCase levels in GBA-PD DA neurons. * *p*-value < 0.05 compared to untreated cells.

The differentiation efficiency was assessed by immunofluorescent staining for markers of DA neurons—Tyrosine hydroxylase (TH) and LIM homeobox transcription factor 1 alpha (LMX1A) (Figure 4A).

In the presence of NCGC607 GCase activity in DA neurons derived from GBA-PD patient was elevated by 1.1-fold (*p* = 0.026) (Figure 4B). Moreover, the same effect of enhanced GCase activity by 1.1-fold we observed in DA neurons derived from controls (*p* = 0.015) (Figure 4B). We observed no differences in HexSph concentration after NCGC607 treatment in DA neurons derived from GBA-PD patients and controls (*p* > 0.05) (Figure 4C).

Also elevated levels of GCase in iPSC-derived DA neurons from patient with GBA-PD (N370S/WT) after NCGC607 treatment was found (*p* = 0.023) (Figure 4D).

## 3. Discussion

Currently, the major challenge in GD is the development of new therapeutic approaches, including disease treatment by PCs, that promote GCase folding and improve its stability and traffic to lysosome. At the same time, the lack of appropriate cellular models that exhibit the accumulation of glycolipids, similar to that observed in patient’s macrophages, has long hampered the development of studies aimed at both finding potential treatments and studying of the GD pathogenesis.

Commonly used fibroblast models of GD lack the characteristic disease phenotype of glycolipid storage in lysosomes. Macrophages are the main cell type exhibiting the disease phenotype [31]. Previously, we and others have showed that macrophages differentiated from PBMCs from patients with GD and GBA-PD characterized by reduced GCase activity and increased accumulation of GlcCer and GlcSph and could be used as a promising tool for assessing an efficiency of GCase PCs [31,34].

In 2016 E. Aflaki and colleagues showed that NCGC607 successfully chaperoned the mutant enzyme, restored GCase activity and protein levels, as well as reduced glycolipid storage both in iPSC-derived macrophages and DA neurons from GD patients. In addition, NCGC607 reduced alpha-synuclein levels in DA neurons from GD patients with parkinsonism, suggesting that NCGC607 or its derivatives may have efficacy as a treatment for PD [25]. In contrast to the study of E. Aflaki and colleagues [25] our research was performed using cultured macrophages. Moreover, in our investigation group of patients with GBA-associated PD was included along with the group of GD patients.

In our study, for the first time we assessed the efficiency of NCGC607 treatment on GCase activity and protein levels, as well as glycolipids concentration in GD and GBA-PD cultured macrophages and described the binding sites of NCGC607 on the GCase surface. The action on GCase activity and protein levels, as well as glycolipids concentration in iPSCs- derived DA neurons from GBA-PD patient with N370S/WT genotype were assessed as well. After NCGC607 treatment restoring GCase activity and protein levels, reducing substrate concentration in GD cultured macrophages were shown. Interestingly, in agreement with our data in the study of E. Aflaki and colleagues NCGC607 treatment is also resulted in significantly enhanced GCase activity and protein levels as well as reduced GCase substrate levels in iPSC-derived macrophages from GD patients [25]. In addition to mentioned above data demonstrated for the first time that NCGC607 treatment increased GCase activity in cultured macrophages from GBA-PD patients (with N370S/WT genotype). Increased GCase activity and protein levels were revealed in iPSCs-derived DA neurons from GBA-PD patient with N370S/WT genotype. In addition, we found the different effects of NCGC607 treatment on GCase activity and HexSph concentration in GD cultured macrophages depending on the mutations in the *GBA1* gene, that could be explained by the mutation-dependent structural changes in the GCase [35]. Notably, ambroxol and other PCs have shown mutation-dependent chemical chaperone profiles previously [24,36,37]. Interestingly even a minor increase in enzyme activity obtained by PCs may have an impact on disease pathology and be beneficial for patients as previously shown. It is assumed that threshold GCase activity of approximately 11–15% is sufficient to prevent the storage of GlcCer in GD [38].

As shown earlier NCGC607 does not inhibit GCase activity and is regarded as a non-inhibitory PC [21]. However, the binding sites of NCGC607 on the GCase surface have not been shown yet.

In this work, we discovered six potential sites on the GCase surface that have suitable volume and binding properties to incorporate chemicals, in particular NCGC607. By summarizing the obtained molecular modeling data, we can propose that only three of these found binding sites—BS1, BS2 and BS3—can be considered as potential targets for NCGC607 compound. These three sites are characterized by the lowest binding free energies and stability of compound position and conformation during 50 ns MD simulations. However, BS3 site is located at the side of the enzyme opposing the active center, in Domain 3, which has less close contacts with the catalytic Domain 2 compared to Domain 1. It is unlikely that binding of compound to this domain can influence the catalytic activity of the enzyme. Conversely, BS1 and BS2 sites are located in close proximity to the active center of GCase and binding of small chemicals here possibly can have indirect effect on enzyme catalytic activity. BS1 site is partially formed by the cavity arising from N370S amino acid substitution and is located under the Loop 6, the structural stability of which, according to the literature, affects the catalytic activity of the enzyme [39]. Binding of the compound in this site is able to stabilize the conformation of the Loop 6 and can affect nucleophilic catalytic residue E340. BS2 site is also located near the active site, but far from the position of S370 residue. Binding of the compound in site BS2 potentially can stabilize the microenvironment of the second acid/base catalytic residue E235. Besides, binding of NCGC607 in sites BS1 or BS2 also can cause the rearrangement in important for catalysis active center of hydrogen bonds network.

Our study is the first one where the possible allosteric binding sites of NCGC607 were evaluated at the surface of monomeric GCase. To our knowledge, the stoichiometry and the binding constants/energy of NCGC607 to GCase have not been published yet. To date only two articles [40,41] have been reported structures of GCase complexes with activators bounded in allosteric sites. The binding sites of these compounds are located at the entrance to the enzyme’s active center and they are at the interface between its two subunits in the dimer found in the crystal structure. Although these activators were shown to promote dimerization of GCase. The published structures of enzyme dimers significantly differ from each other (PDB entries 6T13 and 5LVX) that implies the absence of a universal interface in the enzyme dimer. The authors of the publications do not exclude the possibility that dimerization occurs after the binding of the chemical compound to the enzyme monomer. Considering the above, in our study, the search for binding sites was carried out specifically on the enzyme monomer. However, given that at a certain concentration the enzyme is known to be in equilibrium between the monomeric and dimeric form, our study does not exclude the possibility of binding the NCGC607 to GCase dimeric form.

It is worth noting that NCGC607 compound has less efficiency compared to well-known mixed-type chaperone ambroxol, which is currently ongoing clinical trials for GBA-PD (phase 2, NCT05287503) and for neuronopathic forms of GD (phase 2/3: jRCTs061190017, JPRN-UMIN000009392). It should be noted that NCGC607 unlike ambroxol [24] is not binding to the active site of the enzyme and, therefore, despite a less efficiency in increasing GCase activity, could be more promising for treatment due to binding exclusively to the allosteric site, which will ensure selectivity and may facilitate the selection of an effective dose.

Our study has potential limitations. We assessed GCase protein levels in whole-cell lysates. However, the measurement of GCase protein levels in lysosomal enrichment fractions could be more informative. In our study to evaluate the accumulation of glycolipids we measured concentration of HexSph (GlcSph and galactosylsphingosine (GalSph)) by HPLC-MS/MS. In some studies, macrophages were fed with erythrocyte ghosts isolated from patients with GD to enhanced the accumulation of glycolipids in cells [31,39].

## 4. Materials and Methods

### 4.1. Patients

Patients with GD (*n* = 9, mean age 37.3 ± 4.0 years, 44% men), GBA-PD (PD patients with heterozygous mutations in the *GBA1* gene) (*n* = 6, mean age 55.5 ± 3.5 years, 17% men), and healthy volunteers (*n* = 7, mean age 41.3 ± 4.2 years, 42% men) were enrolled in our study.

The diagnosis of GD was confirmed by measuring GCase activity in leukocytes, while the patient’s genotype was established by sequencing of all exons of the *GBA1* gene. Blood samples from GD patients were taken at least 2 weeks after the last ERT infusion.

GD and PD patients as well as individuals of control group were enrolled at the Almazov National Medical Research Center, Pavlov First Saint-Petersburg State Medical University, Institute of the Human Brain of RAS and FSBI Federal Neurosurgical Center. PD diagnosis was confirmed based on published criteria [42]. *GBA1* variants (N370S and L444P) were confirmed in PD patients and excluded in controls by genotyping, as previously described [11].

Demographics and clinical characteristics of studied groups are presented in Table 2. The study was conducted with the informed consent of all participants and with approval of the local ethics committee.

### 4.2. Differentiation of PBMCs to Macrophages

Differentiation to macrophages from freshly isolated PBMCs (obtained by Ficoll density gradient centrifugation) was carried out in RPMI1640 medium (Corning, Glendale, AZ, USA) supplemented with 10% FBS (Corning, Glendale, AZ, USA) using macrophage colony-stimulating factor (M-CSF) (10 ng/mL) (Biolegend, San Diego, CA, USA) and incubated at 37 °C in 5% CO^2^ for 8 days.

### 4.3. Generation of iPSC and Culture Conditions

Generation of iPSCs from patients PBMCs, culturing and their detailed characterization were carried out according to the previously described protocol [43,44].

### 4.4. Differentiation of Patient-Specific iPSCs to DA Neurons

Directed DA-neurons differentiation of iPSCs was carried out on Matrigel-GFR (Corning, Glendale, AZ, USA) coated dishes according to previously published procedures with some modifications [44,45]. On day 11 of differentiation, the cell monolayer was passaged using Accutase (Thermo Fisher Scientific, Waltham, MA, USA). On the next day, BDNF, GDNF, TGFb3 and dbcAMP (all PeproTech, Cranbury, NJ, USA) were added to the growth medium. The cells were passaged weekly. After 30 days, the cells were transferred to new wells at a density of 1–2 × 10^5^ cells/cm^2^ in the medium containing 0.1 µM Compound E (Sigma-Aldrich, Darmstadt, Germany). Immunofluorescent staining of cells for specific markers (antibodies are listed in Table 3) of DA neurons was performed according to the previously described procedure[46]. 60-day-old DA neurons were used for experiments.

### 4.5. Chemical Synthesis of NCGC607

**Figure 5:**
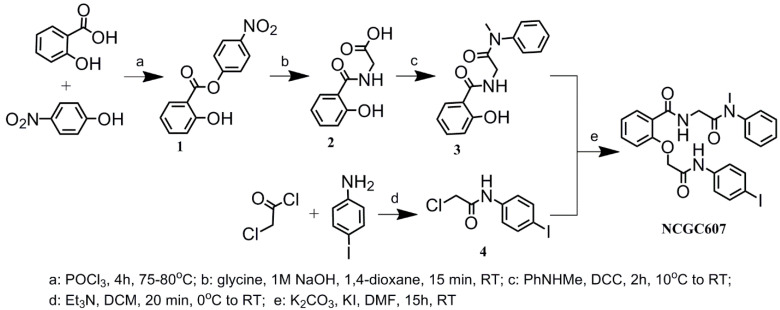
Synthesis of NCGC607.

Synthesis of NCGC607 was performed based on procedure previously described by Aflaki et al. [25] with modifications (Figure 5). Nuclear magnetic resonance (NMR) spectra were recorded with Bruker Avance 400 spectrometer (400.13 MHz for 1H and 100.61 MHz for 13C). Chemical shifts are reported in ppm relative to residual DMSO-d6 (1H, 2.50 ppm), DMSO-d6 (13C, 39.52 ppm) as internal standards. 13C NMR spectra were proton-decoupled. Mass spectra were recorded on a HRMS-ESI-QTOF mass-analyzer, electrospray ionization, positive mode. Melting points were determined on a melting point apparatus and are uncorrected. All chemicals including commercial grade solvents and reagents were purchased from commercial suppliers and used without further purification. Analytical thin layer chromatography (TLC) was performed using Merck 60 F254 silica gel plates to monitor the reaction progress. Chemical synthesis of NCGC607 and its characterization (1H-NMR spectra, 13C-NMR spectra, and HRMS data) are described in detail in Supplementary Description S1.

### 4.6. Chaperone Treatment of Cultured Macrophages and DA Neurons Derived from Patient-Specific iPSCs

To examine the effects of NCGC607 treatment, cultured macrophages and DA neurons derived from patient-specific iPSCs were grown in a complete medium containing 4 µM NCGC607 for 4 and 21 days, respectively. Each line of DA neurons was cultured in 3 wells of a 12 well plate with PCs and 3 wells without PCs (null point) with a daily change of the medium. Chaperone concentration and incubation time were chosen based on previous study [25].

### 4.7. Quantification of GCase Activity and Hexosylsphingosine (HexSph) Concentration

GCase activity and HexSph (GalSph and GlcSph) concentration were measured in triplicate in dry blood and macrophage spots (cell concentration 2 × 10^6^ cell/mL) using high performance liquid chromatography coupled with tandem mass spectrometry (HPLC-MS/MS) as described previously [24,34,47,48]. Whole blood and cell suspensions aliquots were loaded on the filter cards (50 and 20 μL, respectively) (Whatman 903 (Whatman GmbH, Dassel, Germany). Filter cards were dried for 2 h at room temperature and stored at +4 °C until the assays were performed. GCase activity and HexSph concentration were measured in triplicate for each line of DA neurons and normalized to total protein concentration. Briefly, the measurements of GCase activity were performed using the HPLC–MS/MS to determine the concentration of the enzyme reaction product with the substrate. It was assumed that the amount of the product was proportional to the enzyme activity. The protocol of the experiment was based on the technique published by Zhang et al. with modifications [49]. Mix containing substrate, internal standard and extraction buffer solution was added to dried spots of biological material 3.2 mm in diameter. The incubation buffer included 0.1 M ammonium formate buffer (pH 4.4), 8 μmol/L acarbose, and 9.6 g/L sodium taurocholate. The reaction products, internal standards and remaining substrates were separated using the HPLC–MS/MS system that comprised the Shimadzu LC20 HPLC unit (Shimadzu, Kyoto, Japan) and the API 3200 QTrap tandem mass spectrometer (AB Sciex, Framingham, MA, USA) in the mode of multiple reaction monitoring. HexSph concentrations were determined according to the protocol published by Polo et al. with modifications [50]. Lipids were extracted from the spots by addition of 100 μL extraction solvent (80% methanol, 15% acetonitrile, and 5% water) containing 10 ng/mL of internal standard lysolactosylsphingosine (LysoLC) followed by vortexing for 60 min (30 °C, 650 rpm). Extracted lipids were transferred to a new 96-well plate and 10 μL were then injected into the HPLC–MS/MS system consisting of the Shimadzu Nexera HPLC (Shimadzu, Kyoto, Japan) and the API-5500 QTrap mass spectrometer (AB Sciex, Framingham, MA, USA). During chromatographic separation GlcSph and its isomer GalSph elute in a single peak named as HexSph. The MS/MS parameters were optimized for each single standard compound by direct infusion of the solutions (0.1 μmol/L).

### 4.8. Western Blot Analysis

Cells were lysed with a total protein extraction kit (Millipore, Burlington, VT, USA). Total protein concentration was assessed by a Pierce BSA Protein Assay kit (Thermo Fisher Scientific, Waltham, MA, USA). Total protein (20 µg for macrophages and 30 µg for DA neurons) was separated in a 7.5% stain-free gel (Bio-Rad Laboratories, Hercules, CA, USA) with Tris/Glycine/SDS running buffer (Bio-Rad Laboratories, Hercules, CA, USA) and transferred to a PVDF membraine (Bio-Rad Laboratories, Hercules, CA, USA) as previously described [24]. The list of used antibodies is presented in the Table 3. Digital images were obtained by the chemiluminescence system ChemiDoc (Bio-Rad Laboratories, Hercules, CA, USA) and quantified using ImageJ software.

### 4.9. Molecular Modeling

The framework of calculations included the following seven steps: (1) construction of the atomistic models of GCase N370S mutant form and NCGC607 compound, (2) relaxation of the protein structure by 100 ns molecular dynamics simulations in explicit water, (3) cluster analysis of the trajectory and identification of potential binding sites of small molecules on the median structure of the protein from the most populated cluster. Further, for each identified binding site: (4) selection from the MD trajectory of structures representing variety of binding site conformations, (5) 4D docking of NCGC607 molecule using each set of selected conformations (6) selection of three distinct poses of NCGC607 molecule obtained by docking, (7) examination of the stability of each of the three complexes by 100 ns molecular dynamics simulations in explicit water and calculation of the binding free energy of NCGC607 molecule.

#### 4.9.1. Construction of N370S GCase and PC NCGC607 Atomistic Models

Atomistic model of GCase N370S mutant form was prepared for docking using protocol that we described in detail previously [24]. Crude spatial models of NCGC607 (2-[2-(4-iodoanilino)-2-oxoethoxy]-N-[2-(N-methylanilino)-2-oxoethyl] benzamide) molecule were built based on the records of the same name from the PubChem database (https://pubchem.ncbi.nlm.nih.gov; accessed on 19 May 2023) using the Molsoft ICM Pro molecular editor [51]. Geometry of constructed compounds was optimized in Gamess US program [52] using DFT B3LYP method and 3-21G basis set. Water solvent effects were included using the polarizable continuum model. The multiplicity of the electronic state of the molecules was regarded as 1, and the charge state was neutral.

#### 4.9.2. Molecular Dynamics Simulations

Simulations in the explicit solvent were performed as described before [24]. Median structures of the NCGC607 compound from the most populated cluster calculated on a fragment of a trajectory and characterized by the lowest binding free energy were described for each site.

#### 4.9.3. Molecular Docking

Binding sites identification and molecular docking simulations were performed in Molsoft ICM Pro 3.8 program package [51]. PocketFinder module of ICM Pro was used to reveal potential binding sites on surface of GCase. Six sites having the largest volumes were chosen for molecular docking. To take into consideration flexibility of binding sites we generated conformational ensembles of the protein by molecular dynamics simulations. Obtained GCase structures were clustered by positions of amino acid residues forming binding sites. Median structures of each cluster were chosen for ensemble docking procedure called four-dimensional docking (4D docking) [53]. 4D docking accounts for the conformational variability of the receptor in a single docking simulation and reduces the sampling time while preserving the accuracy of traditional ensemble docking.

For each of six binding sites docking results were filtered according to values of scoring function and three distinct poses of NCGC607 compound were manually selected for refinement and stability testing by 50 ns MD simulations.

### 4.10. Statistical Analysis

Statistical analyses were performed using SPSS (version 21.0; USA) software. All results were tested for a normal distribution using the Kolmogorov–Smirnov test and the Shapiro–Wilk test. Significance was determined by the Wilcoxon signed rank test. Clinical data are presented as mean ± standard error of the mean (SEM) values. Experimental data are presented as median of values. The level of significance was set at *p* < 0.05.

## 5. Conclusions

Our results showed the influence of NCGC607 on GCase activity in cultured cells from GD and GBA-PD patients and confirmed its efficacy on iPSC-derived DA neurons from GBA-PD patient. Molecular modeling of NCGC607 binding to GCase demonstrated that this compound could act as an allosteric PC.

## Figures and Tables

**Table 1:** Characteristics of GCase potential binding sites. Described residues used in molecular docking for binding site definition.

Site	Residues	Binding Free Energy, kcal/mol
BS1	S366, T369, S370, Q362, G344, V343, W312, D315, F316, A318, P319, A320	−53.7 ± 4.0
BS2	K194, G195, S196, L197, G199, Q200, G239, L249, G250, F251, T252, H255	−48.7 ± 2.5
BS3	E429, P428, I427, F426, K425, P29, R170, H451, S455	−35.3 ± 2.3
BS4	G64, L65, D467, V468, D443, D444, N442, K441, Q440, S439	−32.1 ± 2.5
BS5	H274, V276, R277, L278, H306, K303, Y304, V305, G265, L268, A269	−34.3 ± 3.2
BS6	F128, S129, I130, R131, T132, N192, P391	−29.00 ± 2.9

**Table 2:** Demographics and clinical data of the GD, GBA-PD patients and controls.

Patients	GD Type	Type of Therapy	Sex	Age, Years	Mutation in the *GBA1* Gene	GCase Residual Activity in Blood, %	HexSph Concentration in Blood, %
GD 1	1	ERT	m	55	N370S/c84dupG	0.4	6795
GD 2	1	ERT	f	48	N370S/L444P	4.74	16,216
GD 3	1	SRT	f	20	N370S/L444P	9.3	13,281
GD 4	1	ERT	f	31	N370S/L444P	3.2	15,212
GD 5	1	ERT	m	50	N370S/-	4.8	1738
GD 6	1	ERT	f	35	N370S/R120W	2.8	11,003
GD 7	1	ERT	m	29	N370S/L444P	5.9	27,413
GD 8	1	-	m	26	N370S/R120W	10.3	45,637
GD 9	1	ERT	f	42	N370S/W184R	5.1	21,042
GBA-PD 1	-	L-DOPA	f	62	WT/L444P	52.6	320
GBA-PD 2	-	L-DOPA	f	50	WT/L444P	50.1	192
GBA-PD 3	-	L-DOPA	m	42	N370S/WT	78.8	294
GBA-PD 4	-	L-DOPA	f	66	N370S/WT	54.7	219
GBA-PD 5	-	L-DOPA	f	57	N370S/WT	55.6	357
GBA-PD 6	-	L-DOPA	f	56	N370S/WT	38.1	201
Controls (*n* = 7)	-	-	42% men	41.3 ± 4.2	WT/WT	100	100

GD—Gaucher disease, GBA-PD—Parkinson’s disease associated with mutation in the *GBA1* gene, ERT—enzyme replacement therapy, SRT—substrate reduction therapy, L-DOPA—L-3,4-dihydroxyphenylalanine (Levodopa), WT—wild type, GCase—glucocerebrosidase, HexSph—hexosylsphingosine.

**Table 3:** List of used antibodies.

Antibodies	Company	Cat. Ref.	Raised/Type	Dilution
Primary antibodies				
Anti-TH	Millipore, Burlington, VT, USA	AB152	IgG rabbit polyclonal	1:400
Anti-LMX1A	Abcam, Cambridge, UK	ab139726	IgG rabbit polyclonal	1:100
Anti-TUJ1	Covance, Princeton, NJ, USA	MMS-435P	IgG2a mouse monoclonal	1:1000
Anti-GBA	Abcam, Cambridge, UK	Ab125065	IgG rabbit monoclonal	1:500
Anti-GAPDH	Sigma-Aldrich, Darmstadt, Germany	SAB2108266	IgG rabbit polyclonal	1:18000
Secondary antibodies				
Alexa Fluor 488 goat anti rabbit IgG (H + L)	Thermo Fisher Scientific, Waltham, MA, USA	A11008	Goat	1:400
Alexa Fluor 568 goat anti rabbit IgG (H + L)	Thermo Fisher Scientific, Waltham, MA, USA	A11011	Goat	1:400
Alexa Fluor 488 goat anti mouse IgG2a	Thermo Fisher Scientific, Waltham, MA, USA	A21131	Goat	1:400
Goat Anti-Rabbit IgG H&L (HRP)	Abcam, Cambridge, UK	Ab6721	Goat	1:5000

## Data Availability

Characterization of iPSCs are presented in the Human Pluripotent Stem Cell Registry (hPSCreg): for GBA-PD (https://hpscreg.eu/cell-line/ICGi034-A; https://hpscreg.eu/cellline/ICGi034-B; https://hpscreg.eu/cell-line/ICGi034-C; all accessed on 19 May 2023).

## Supplementary Materials

The following supporting information can be downloaded at: https://www.mdpi.com/article/10.3390/ijms24109105/s1.
