# Supplementary material for: Potential Binding Sites of Pharmacological Chaperone NCGC00241607 on Mutant β-Glucocerebrosidase and Its Efficacy on Patient-Derived Cell Cultures in Gaucher and Parkinson’s Disease
Source: Int J Mol Sci. 2023 May 22;24(10):9105. doi: 10.3390/ijms24109105 (PMC10219579; doi:10.3390/ijms24109105)
Supplement: Supplementary file 1 [file ijms-24-09105-s001.zip › Description S1.pdf]

Synthesis of NCGC607

NMR spectra were recorded with Bruker Avance 400 spectrometer (400.13 MHz for ^{1}H and 100.61 MHz for ^{13}C). Chemical shifts are reported in ppm relative to residual DMSO-d_6 (^{1}H, 2.50 ppm), DMSO-d_6 (^{13}C, 39.52 ppm) as internal standards. ^{13}C NMR spectra were proton-decoupled. Mass spectra were recorded on a HRMS-ESI-QTOF mass-analyzer, electrospray ionization, positive mode. Melting points were determined on a melting point apparatus and are uncorrected. All chemicals were purchased from commercial suppliers and used without further purification. Commercial grade solvents and reagents were used without further purification. Analytical thin layer chromatography (TLC) was performed using Merck 60 F254 silica gel plates to monitor the reaction progress. Synthesis of NCGC607 was performed based on previously described by Sidransky et al. [15] procedure and modified by us as followed (Figure X):

*4-nitrophenyl 2-hydroxybenzoate (**1**)*

Phosphoryl chloride (7.66 mL, 84 mmol) was added to a mixture of salicylic acid (27.62 g, 200 mmol) and 4-nitrophenol (27.82 g, 200 mmol). The reaction mixture was stirred for 4 h at 75 – 80□C, cooled to room temperature and poured into suspension of sodium bicarbonate (26.90 g, 320 mmol) in water (175 ml). The obtained precipitate was filtered off, washed successively with water and dried to give 34.7 g of 4-nitrophenyl 2-hydroxybenzoate as a grayish crystals in 67% yield. The compound was used for the next reaction step without further purification.

*2-(2-hydroxybenzamido)acetic acid (**2**)*

A solution of glycine (4.50 g, 60 mmol) in 1M sodium hydroxide (60 mL) was added to the suspension of 4-nitrophenyl 2-hydroxybenzoate (12.96 g, 50 mmol) in 1,4-dioxane (70 mL) and the reaction mixture was stirred for 15 h at room temperature and then extracted twice with dichloromethane to remove 4-nitrophenol. Combined extracts were acidified to pH 2, washed successively with water and dried to give 6.35 g of 2-(2-hydroxybenzamido)acetic acid as a crystals in 65% yield. The obtained product was used without further purification.

*2-hydroxy-N-(2-(methyl(phenyl)amino)-2-oxoethyl)benzamide (**3**)*

Dicyclohexylcarbodiimide (3.40 g, 17 mmol) in ethylacetate (15 mL) was added under stirring to a cooled to 10 □C mixture of 2-(2-hydroxybenzamido)acetic acid (2.93 g, 15 mmol) and *N*-methylaniline (1.95 mL,18 mmol) in acetonitrile (15 mL). The reaction mixture was stirred for 2 h at room temperature. Precipitated dicyclohexylurea was filtered off, solvent was evaporated under reduced pressure. Crude residue was crystallized under hexane and recrystallized from hexane – ethyl acetate mixture to give 3.37 g of 2-hydroxy-*N*-(2-(methyl(phenyl)amino)-2-oxoethyl)benzamide as colorless crystals. The yield was 3.37 g (79%).

*2-chloro-N-(4-iodophenyl)acetamide (**4**)*

Chloroacetyl chloride (7.57 mL, 96 mmol) was added dropwise to a stirred cold (0^0C) mixture of 4-iodoaniline (12.52 g, 80 mmol) and triethylamine (14.5 mL, 104 mmol) in anhydrous dichloromethane (160 mL) at 0□C under stirring. The reaction mixture was stirred for 20 min at room temperature, than washed with ice water (2x100 mL) and dried over anhydrous magnesium sulfate. The solvent was removed under reduced pressure to give 2-chloro-*N*-(4-iodophenyl)acetamide as colorless crystals. The yield was 20.57 g (87%).

*2-(methyl(phenyl)amino)-2-oxoethyl 2-(2-((4-iodophenyl)amino)-2-oxoethoxy)benzoate (**NCGC607**)*

To a solution of 2-hydroxy-*N*-(2-(methyl(phenyl)amino)-2-oxoethyl)benzamide (2.84 g, 10 mmol), 2-chloro-*N*-(4-iodophenyl)acetamide (2.96 g, 10 mmol) and potassium iodide (83 mg, 10 mmol) in DMF (20 mL) unhydrous powdered potassium carbonate (1.38 g, 10 mmol) and reaction mixture was stirred for 15 h at room temperature. The product was isolated by adding an equal volume of water, followed by filtration. The resulting precipitate was washed successively with water and then with isopropyl alcohol. Crude product was recrystallized from DMF – ethyl acetate mixture to give 4.24 g of 2-(methyl(phenyl)amino)-2-oxoethyl 2-(2-((4-iodophenyl)amino)-2-oxoethoxy)benzoate as a colorless crystals. The yield was 78%.

^{1}H NMR (400 MHz, DMSO-d$_6$): δ = 10.44 (s, 1H), 8.85 (t, J = 5.1 Hz, 1 H), 7.73 (d, J = 7.2 Hz, 1 H), 7.68 (d, J = 8.6 Hz, 1 H), 7.56–7.38 (m, 8 H), 7.17 (d, J = 8.3 Hz, 1 H), 7.09 (t, J = 7.5 Hz, 1 H),

4.90 (s, 2 H), 3.83 (br. s, 2 H), 3.20 (br. s, 3 H). ^{13}C NMR (101 MHz, DMSO-d_6): δ = 167.1, 166.0, 156.2, 138.6, 137.9, 133.0, 130.8, 123.7, 122.2, 122.0, 114.3, 87.9, 68.3, 42.4, 37.5. HRMS-ESI: *m*/*z* $[M + Na]^+$ calcd for $C_{24}H_{22}IN_3O_4Na^+$: 566.0547; found: 566.0560.
