# Supplementary figures and images for: Potential Binding Sites of Pharmacological Chaperone NCGC00241607 on Mutant β-Glucocerebrosidase and Its Efficacy on Patient-Derived Cell Cultures in Gaucher and Parkinson’s Disease
Source: Int J Mol Sci. 2023 May 22;24(10):9105. doi: 10.3390/ijms24109105 (PMC10219579; doi:10.3390/ijms24109105)

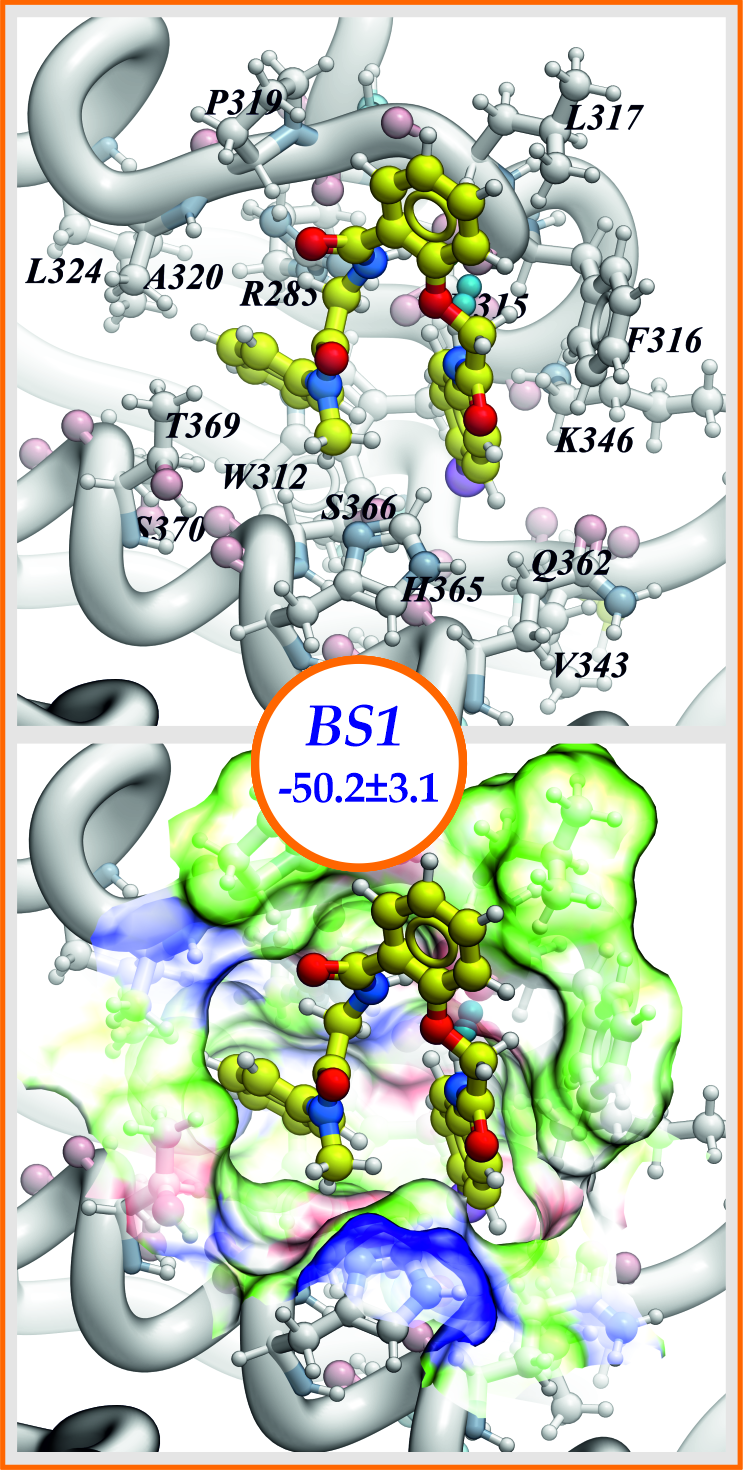

Supplement: Supplementary file 1 [file ijms-24-09105-s001.zip › Figure S1.tif]

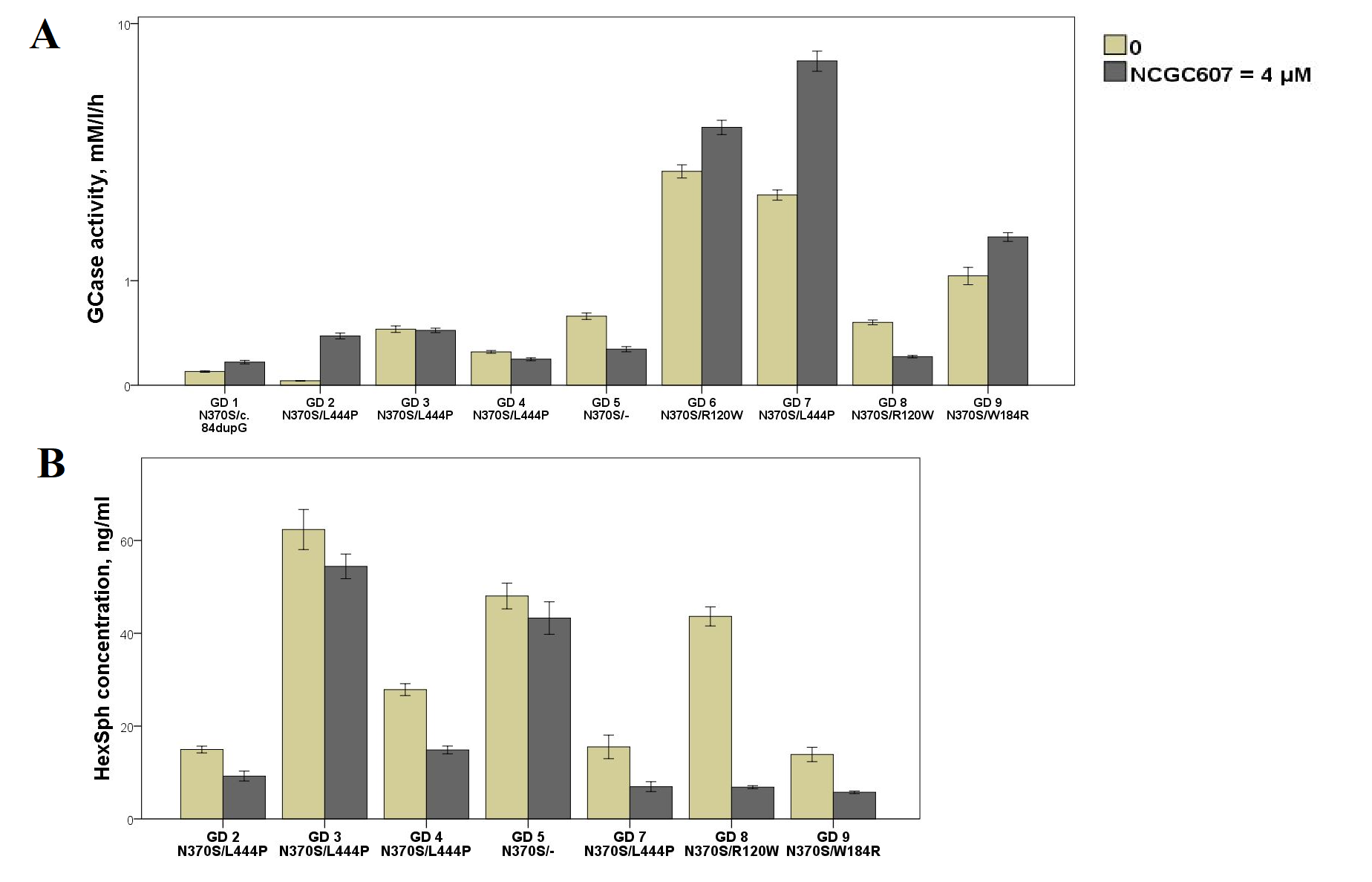

Supplement: Supplementary file 1 [file ijms-24-09105-s001.zip › Figure S2.tif]
